# Evaluation of concordance of new QuantiFERON-TB Gold Plus platforms for *Mycobacterium tuberculosis* infection diagnosis in a prospective cohort of household contacts

**DOI:** 10.1128/spectrum.00469-24

**Published:** 2024-07-08

**Authors:** Cinthya Ruiz-Tagle, Patricia García, Mariluz Hernández, María Elvira Balcells

**Affiliations:** 1 Departamento de Enfermedades Infecciosas del Adulto, Escuela de Medicina, Pontificia Universidad Católica de Chile, Santiago, Chile; 2 Departamento de Laboratorios Clínicos, Escuela de Medicina, Pontificia Universidad Católica de Chile, Santiago, Chile; 3 Equipo Técnico de Tuberculosis, Dirección de Servicio de Salud Oriente, Santiago, Chile; 4 Red de Salud UC-CHRISTUS, Santiago, Chile; Duke University, Durham, North Carolina, USA

**Keywords:** *Mycobacterium tuberculosis*, tuberculosis, QuantiFERON, interferon-gamma release assay, diagnostic test, latent tuberculosis infection

## Abstract

Interferon-gamma (IFN-γ) release assays play a pivotal role in tuberculosis infection (TBI) diagnosis, with QuantiFERON-TB Gold Plus—an enzyme-linked immunosorbent assay (ELISA)—among the most widely utilized. Newer QuantiFERON-TB platforms with shorter turnaround times were recently released. We aimed to evaluate these platforms’ agreement in the diagnosis of TBI. Blood samples from a prospective cohort of tuberculosis household contacts were collected at baseline and after 12 weeks of follow-up, and tested with LIAISON, an automated chemiluminescence immunoassay (CLIA) system, QIAreach, a lateral flow (QFT-LF) semi-automated immunoassay, and the ELISA QuantiFERON-TB Gold Plus platform. Test concordances were analyzed. ELISA vs CLIA overall agreement was 83.3% for all tested samples (120/144) [Cohen’s kappa coefficient (κ): 0.66 (95% CI: 0.54–0.77)]. Samples positive with CLIA provided consistently higher IFN-γ levels than with ELISA (*P* < 0.001). Twenty-four (16.7%) discordant pairs were obtained, all CLIA-positive/ELISA-negative: 15 (62.5%) had CLIA IFN-γ levels within borderline values (0.35–0.99 IU/mL) and 9 (37.5%) >0.99 IU/mL. QFT-LF showed only 76.4% (68/89) overall agreement with ELISA [κ: 0.53 (95% CI: 0.37–0.68)] with 21 (23.6%) discordant results obtained, all QFT-LF-positive/ELISA-negative. Overall concordance between ELISA and CLIA platforms was substantial, and only moderate between ELISA and QFT-LF. The CLIA platform yielded higher IFN-γ levels than ELISA, leading to an almost 17% higher positivity rate. The techniques do not seem interchangeable, and validation against other gold standards, such as microbiologically-confirmed tuberculosis disease, is required to determine whether these cases represent true new infections or whether CLIA necessitates a higher cutoff.

**IMPORTANCE:**

Tuberculosis is an airborne infectious disease caused by *Mycobacterium tuberculosis* that affects over 10 million people annually, with over 2 billion people carrying an asymptomatic tuberculosis infection (TBI) worldwide. Currently, TBI diagnosis includes tuberculin skin test and the blood-based interferon-gamma (IFN-γ) release assays, with Qiagen QuantiFERON-TB Gold Plus (QFT) being among those most widely utilized. We evaluated Qiagen's newer QFT platforms commercially available in a prospective cohort of tuberculosis contacts. A substantial agreement was obtained between the current QFT-enzyme-linked immunosorbent assay (ELISA) and the new QFT-chemiluminescence immunoassay (CLIA) platform, although QFT-CLIA provided higher concentrations of IFN-γ, leading to a 16.6% higher positivity rate. We highlight that both platforms may not be directly interchangeable and that further validation is required.

## INTRODUCTION

Tuberculosis (TB) is the second leading infectious killer after COVID-19. In 2022, 10.6 million people fell ill with TB worldwide and 1.3 million people died (1). Currently, the main tools for *Mycobacterium tuberculosis* infection (TBI) diagnosis in contact with patients with TB disease include tuberculin skin tests and interferon-gamma (IFN-γ) release assays (IGRAs). In 2011, the World Health Organization (WHO) issued recommendations on the use of IGRAs for the diagnosis of TBI, including the blood-based Qiagen QuantiFERON-Gold, QuantiFERON-TB Gold In-Tube, and Oxford Immunotec T-SPOT.TB assays (2). Based on new and updated versions of the marketed blood-based IGRAs, the WHO recently updated its recommendations adding Beijing Wantai’s TB-IGRA and Qiagen QuantiFERON-TB Gold Plus (QFT), which have comparable performance to that of previously WHO-recommended IGRAs (3).

In 2018, Qiagen in alliance with Diasorin released the LIAISON, a new fully automated chemiluminescence immunoassay (CLIA) platform for the already existing QFT, previously only performed as an enzyme-linked immunosorbent assay (ELISA). This new version is currently marketed in several countries, including Chile. A high degree of concordance for interpretative results between the two techniques (92%–97.5%) (4–9) was reported in six studies, while another showed only substantial agreement (75.4%) (10). Although the overall correlation between TB1 and TB2 tubes for both techniques was strong, the majority of these studies have also reported that QFT-CLIA yields higher concentrations of IFN-γ (5, 6, 8–10). None of these studies were conducted in recent TB contacts.

Additionally, in 2021, Qiagen in alliance with Ellume Ltd. released the QIAreach QuantiFERON-TB (QFT-LF), a lateral flow semi-automated immunoassay that uses only one tube with antigens (TB2), does not require an established laboratory infrastructure or trained personnel, and provides a final qualitative result within 20 minutes of reading (11, 12). Its implementation suitability in remote areas and with limited infrastructure was demonstrated (13). Nonetheless, although early reports showed a high agreement with QFT-ELISA (94.9%–98.8%) in low and intermediate incidence settings, others showed inconsistent results regarding the correlation between the time to result and IFN-γ concentrations when levels were below or near the manufacturer’s cutoff for positivity (0.35 IU/mL) (11, 12, 14, 15). As of November 2022, its commercialization has been paused (3).

In the present study, we compared the agreement between QFT-ELISA (as the Gold Standard), QFT-CLIA, and QFT-LF platforms in the study of TBI in a prospective cohort of TB household contacts to assess their interchangeability in the diagnosis of TBI.

## RESULTS

### Study participants characteristics

#### QFT-ELISA vs QFT-CLIA

The TB household contacts included in this analysis (*n* = 117) were 55.6% females, with a median age of 29 years (range, 6–95 years), 23.9% children between 6 and 17 years, and 56.4% migrants of which 51.5% were from countries with a medium or high TB incidence (Bolivia, Peru, and Haiti). None of them had HIV infection; one participant had cancer (0.9%) and three had diabetes mellitus (2.6%). Most participants (98.3%) have been previously vaccinated with BCG in the past.

#### QFT-ELISA vs QFT-LF

TB household contacts included in this analysis (*n* = 66) were 53% females, with a median age of 29.5 years (range, 6–90 years), 30.3% children between 6 and 17 years, and 36.4% migrants of which 62.5% were from countries with a medium or high TB incidence (Bolivia, Peru, Haiti, and Morocco). None of them presented an immunosuppressive condition; four participants had diabetes mellitus (6.1%). Most participants (90.9%) had been previously vaccinated with BCG in the past.

### Comparison between QFT-ELISA and QFT-CLIA results

**Fig 1:**
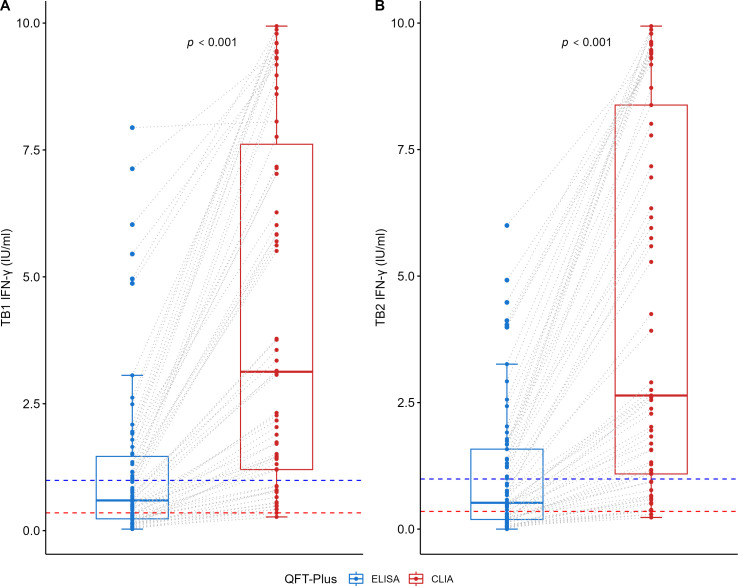
Comparison of IFN-γ levels obtained with simultaneous QFT-ELISA and QFT-CLIA in fresh samples (only QFT-CLIA-positive results included). (A) TB1 (*n* = 66) and (B) TB2 (*n* = 65). The red and blue dotted lines correspond to the borderline values 0.35 and 0.99 IU/mL, respectively. Comparisons were performed using the Wilcoxon test for paired samples; for analyses, two-sided *P*-values < 0.05 were considered statistically significant.

In total, 145 fresh samples were simultaneously analyzed by QFT-ELISA and QFT-CLIA platforms. One sample was QFT-CLIA-negative and QFT-ELISA-indeterminate and therefore was excluded from the analysis. From the remaining 144 samples, 29.9% resulted positive by QFT-ELISA and 46.5% by QFT-CLIA (Table 1). There was an 83.3% [120/144 (95% CI: 77.2%–89.4%)] overall agreement between both tests [Cohen’s kappa coefficient: 0.66 (95% CI: 0.54–0.78)]. Overall, significantly higher levels of IFN-γ were observed with QFT-CLIA results compared with QFT-ELISA’s. For all positive results the median IFN-γ values for QFT-ELISA and QFT-CLIA were 0.60 vs 3.13 IU/mL (*n* = 66, *P* < 0.001) for TB1, and 0.52 vs 2.64 IU/mL (*n* = 65, *P* < 0.001) for TB2, respectively (Fig. 1). For the negative QFT results, significantly different results were observed for TB2 only (Fig. S1). As a complementary analysis, we plotted IFN-γ differences between QFT-CLIA and QFT-ELISA measurements for all samples; median differences were 2.33 IU/mL (*n* = 61) and 2.24 IU/mL (*n* = 61) for TB1 and TB2, respectively (Fig. S2).

**TABLE 1:** QFT-ELISA and QFT-CLIA qualitative agreement for fresh samples (*n* = 144)

		QFT-CLIA	
		Positive	Negative
**QFT-ELISA**	**Positive**	43 (100%)	0 (0%)
	**Negative**	24 (23.8%)	77 (76.2%)

Quantitative discordance for ELISA-negative/CLIA-positive		
TB1-CLIA	Below 0.35 IU/mL	*n* = 1
	Between 0.35 and 0.99 IU/mL	*n* = 14
	Above 0.99 IU/mL	*n* = 9
TB2-CLIA	Below 0.35 IU/mL	*n* = 3
	Between 0.35 and 0.99 IU/mL	*n* = 12
	Above 0.99 IU/mL	*n* = 9

**Fig 2:**
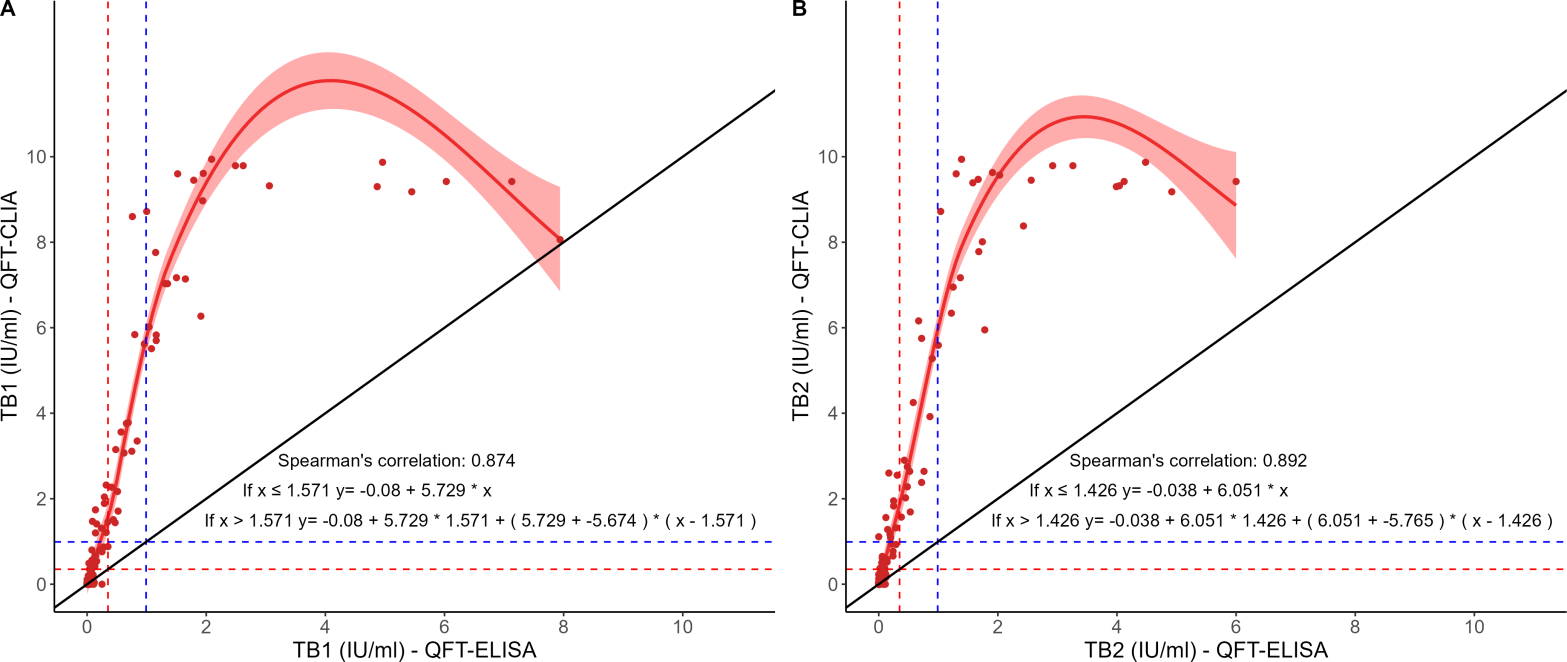
Comparison of IFN-γ levels obtained with simultaneous QFT-ELISA and QFT-CLIA in all fresh samples. (A) TB1 and (B) TB2. The red and blue dotted lines correspond to the borderline values 0.35 and 0.99 IU/mL, respectively. The red line corresponds to the segmented regression line and the light red area to its confidence interval. The black line corresponds to the line of equality. Inside the plot are Spearman’s correlation coefficient and the equations for the segmented regression, where x corresponds to the value obtained with QFT-ELISA.

**Fig 3:**
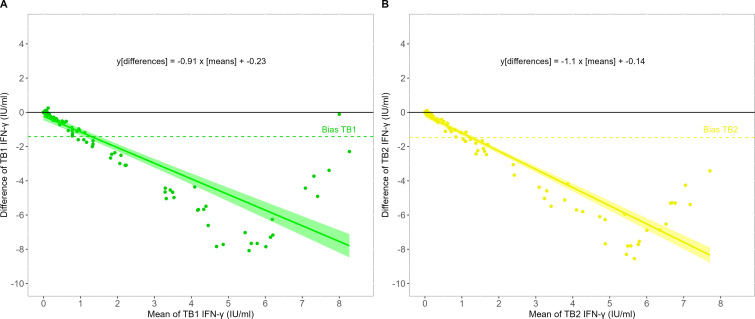
Bland-Altman plots showing the agreement of IFN-γ levels measured with QFT-ELISA and QFT-CLIA in all fresh samples. Results for (A) TB1 and (B) TB2. The y-axis depicts the difference between IFN-γ levels obtained with QFT-ELISA minus those obtained with QFT-CLIA, while the x-axis depicts the mean IFN-γ levels obtained with QFT-ELISA and QFT-CLIA. The dotted line corresponds to the bias and the black line to the line of equality. Inside the plot is the calculated regression line.

A total of 24 (16.7%) discordant paired test results were obtained, of which 15 were QFT-CLIA-positive with either TB1 or TB2 IFN-γ values within the borderline zone, and nine with values above 0.99 IU/mL (from 1.20 to 2.32 IU/mL for TB1, and from 1.09 to 2.60 IU/mL for TB2). There were no QFT-CLIA-negative/QFT-ELISA-positive discordant results. Among individuals with discordant results, nine were re-sampled after 3 months. Of them, all but one resulted again positive with QFT-CLIA, and all but one resulted negative with QFT-ELISA. A non-linear positive correlation between TB1 [r_S_ = 0.87 (95% CI: 0.83–0.91)] and TB2 [r_S_ = 0.89 (95% CI: 0.85–0.92)] measured with both techniques was found (Fig. 2). The Bland-Altman plot, which allows the comparison of two measurement techniques on the same quantitative variable, showed that both methods produced different results (values far from zero), with QFT-CLIA yielding higher values than the currently validated method. The bias for TB1 and TB2, calculated as QFT-ELISA minus QFT-CLIA, was −1.419 IU/mL (95% CI, −1.800 to −1.037) and −1.478 IU/mL (95% CI, −1.875 to −1.081), respectively, and this significant negative bias was observed due to measurements over 1 IU/mL (Fig. 3).

For a subset of these samples (*n* = 70), we further assessed whether temporal storage (3 months at −80°C) would affect IFN-γ level measurements for both platforms. The positivity by QFT-ELISA decreased from 22.9% (16/70) to 17.1% (12/70), while the 38.6% (27/70) positivity by QFT-CLIA remained unaffected. On the other hand, the comparison of median IFN-γ levels for paired analysis of fresh vs frozen QFT-positive samples showed no significant differences between both platforms, except a minimal drop for TB1 median [1.71 vs 1.55 IU/mL (*n* = 27, *P* = 0.009)] with QFT-CLIA (Fig. S3).

**Fig 4:**
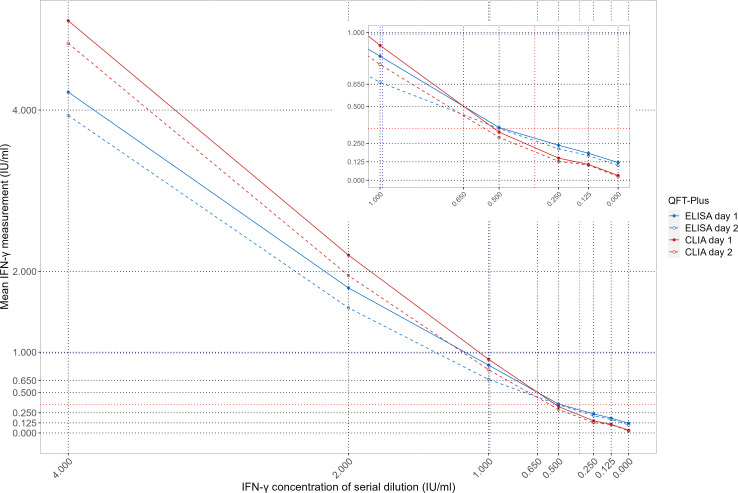
Measurements obtained with QFT-ELISA and QFT-CLIA for a 7-point human IFN-γ standard curve. Two experiments were performed to construct a 7-point IFN-γ standard curve (4, 2, 1, 0.5, 0.25, 0.125, and 0 IU/mL). Each point represents the average of a triplicate measurement with both QFT-ELISA and QFT-CLIA. The zoomed zone corresponds to a section from the 7-point curve (1, 0.5, 0.25, 0.125, and 0 IU/mL).

Additionally, two separate assays were performed in triplicate to determine concentrations in a 7-point curve of serial dilution of IFN-γ standard preparations. Based on the plot, we observed an inflection point at 0.65 IU/mL, from which QFT-CLIA measurements showed a non-significant trend toward higher IFN-γ levels compared to QFT-ELISA (for trend *P* = 0.42) (Fig. 4).

### Comparison between QFT-ELISA and QFT-LF

**Fig 5:**
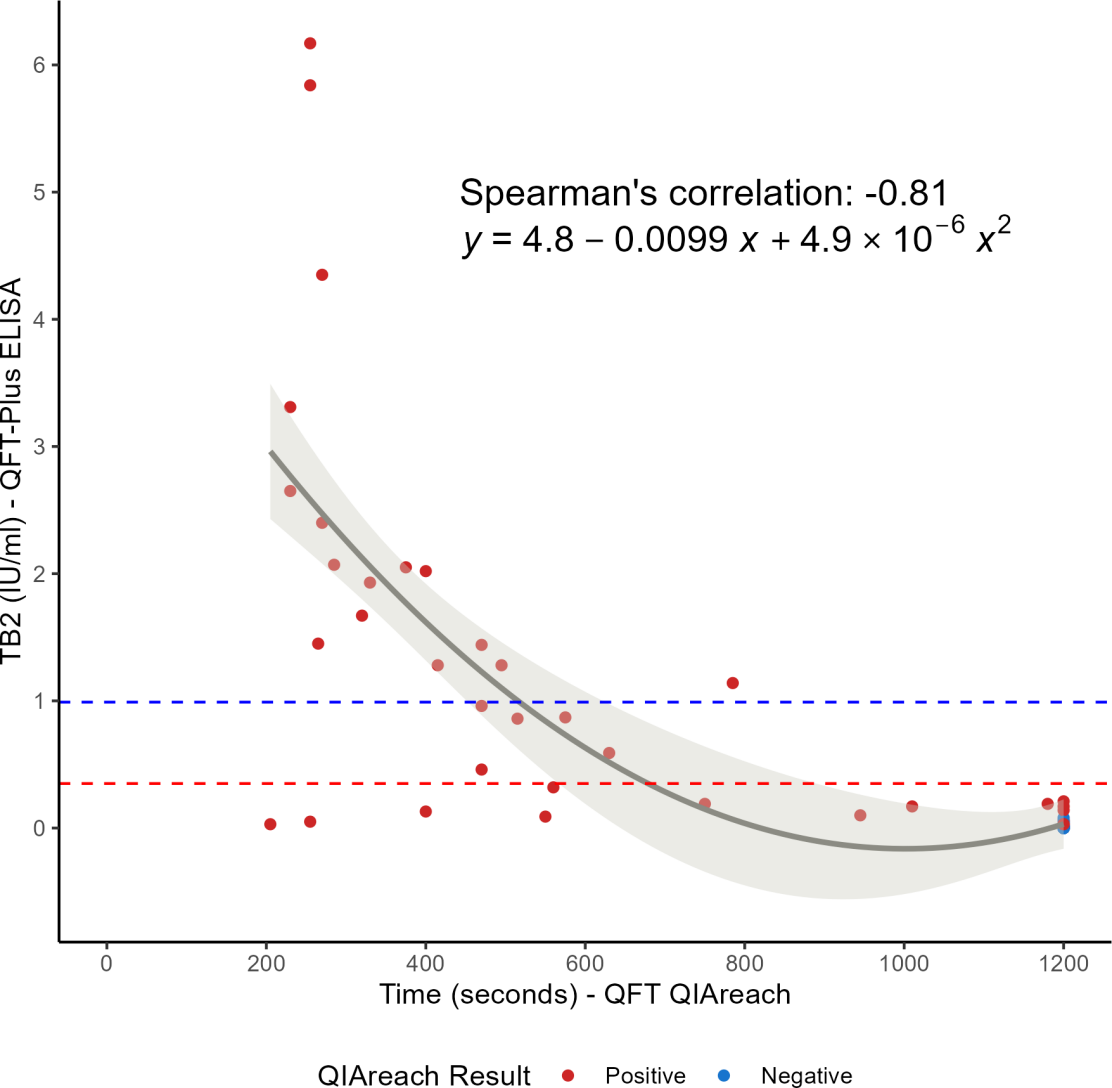
IFN-γ levels obtained with QFT-ELISA vs the time to result for QFT-LF. The red and blue dotted lines correspond to the borderline values 0.35 and 0.99 IU/mL, respectively. The ivory line corresponds to the polynomial regression line and the light ivory area to its confidence interval. Inside the plot are the Spearman’s correlation coefficient and the polynomial equation. The red and blue dots correspond to QFT-LF positive and negative results, respectively.

In total, 89 samples were analyzed, obtaining a 76.4% (68/89) overall agreement between QFT-LF and QFT-ELISA platforms [Cohen’s kappa coefficient: 0.53 (95% CI: 0.37–0.68)]. For qualitative results, 25.8% (*n* = 23) resulted positive by QFT-ELISA and 49.4% (*n* = 44) positive by QFT-LF (Table 2). All discordant results were QFT-LF-positive/QFT-ELISA-negative (*n* = 21, 23.6%), of which 14 reached positivity at QFT-LF time to result (TTR) of 1,200 seconds (20 minutes, maximum time) (Fig. 5). We found a negative correlation between TB2 IFN-γ levels of QFT-ELISA and the QFT-LF TTR [r_S_ = −0.81 (95% CI of −0.88 to −0.73)]. Regarding QFT TB2 positive only (*n* = 21, 23.6%), positivity by QFT-LF was also 49.4% (*n* = 44).

**TABLE 2:** QFT-ELISA and QFT-LF qualitative agreement (*n* = 144)

		QFT-LF	
		Positive	Negative
**QFT-ELISA**	**Positive**	23 (100%)	0 (0%)
	**Negative**	21 (31.8%)	45 (68.2%)

## DISCUSSION

Our results show that the QFT-CLIA platform reports consistently higher IFN-γ values than QFT-ELISA for both TB1 and TB2, translating into a 16.6% higher positivity rate for QFT-CLIA within this cohort of TB contacts, with most of the samples being within the borderline range of positivity (0.35–0.99 IU/mL). The proportion of overall discordant results (23.8%) was higher than in previous reports in which discordance between both tests ranged from 2.55% to 8% only (4–9). With the current study design, it is not possible to conclude whether this 16.6% extra positivity corresponds to true TBI (that may go undetected by QFT-ELISA) or to QFT-CLIA false-positive results due to a higher level of IFN-γ reporting. It is known that QFT-ELISA may miss cases of recently acquired TBI, as seen in our prior study in which six household contacts without immunosuppressive conditions initially tested negative but developed active TB within 3 months (16). However, we found that resampling the TB contacts after 3 months did not change the direction of the results in 8 out of 9 QFT-ELISA-negative/QFT-CLIA-positive discordant pairs. Therefore, it is unlikely that the discrepancy could rely on a longer latency of QFT-ELISA to detect TBI converters vs QFT-CLIA.

We also observed measurement discrepancies in the analysis of the 7-point IFN-γ standard curve, in which a non-significant trend toward higher QFT-CLIA measurements for IFN-γ concentrations >0.65 IU/mL was observed. This supports the idea that a calibrator curve might not be the answer, as already mentioned by others (9). Moreover, our findings are consistent with recent studies that identified a TB1 and TB2 response of 0.35 to 1.55 IU/mL, and 0.35 to 1.45 IU/mL, respectively, as the range for non-reproducible positive QFT-CLIA results (9). The range for non-reproducibility in our study was broader [TB1 (0.35 to 2.32 IU/mL) and TB2 (0.35 to 2.60 IU/mL)]. The methodology used might account for these differences; our study analyzed fresh samples manually and simultaneously, while the other one used an automated robotic system and included 15.5% of samples being frozen for months before being tested with QFT-CLIA.

The overestimation of QFT-CLIA measurements has been described by others and initially was attributed to pre-analytical factors (5, 6, 10), as well as to the detection method itself (9). Either way, it is of the utmost importance that the manufacturer determines the nature of these discrepancies (i.e., intrinsic or extrinsic to the chemistry, the technique, or the patient’ sample itself) to rule out false-positive reporting. Additionally, the higher positivity rate obtained with QFT-CLIA should be validated against other gold standards, such as TB disease, as it was done for QFT-ELISA before widespread implementation (17). Otherwise, there may be a direct and non-dismissible consequence in over-diagnosing TBI, particularly in the current era in which the WHO supports TBI investigation in all household contacts, which may entrain higher operational costs for national TB programs and confer the unnecessary risk from overtreatment individuals (18).

Confirming borderline-positive results with QFT-ELISA has been proposed as a viable strategy to mitigate false-positive QFT-CLIA results (9). However, this would translate into the need to repeat testing with higher costs (QFT-ELISA instrument and personnel) and delays in laboratory results. Moreover, if these additional positive results with QFT-CLIA are indeed true infections, this alternative may be risky. Thus, revising and modifying the QFT-CLIA manufacturer’s interpretive criteria with the current cutoff, as suggested (5, 6, 8, 10), requires an independent previous validation accuracy study. A meta-analysis assessing the diagnostic performance of new commercial IGRAs, included only four studies comparing QFT-ELISA vs QFT-CLIA with an 88.6% global concordance (1,039/1,173), a pooled agreement of 0.86 (95% CI 0.78–0.94) with a low certainty of the evidence, supporting the need for further studies (19). Only one small study included samples from patients with microbiologically confirmed active TB (*n* = 15), showing a QFT-CLIA positivity of 73% and a 92.8% agreement between QFT-CLIA and QFT-ELISA (7). In this same study, the authors found a 3.9% QFT-CLIA positivity in participants with no identified risk factors for TB infection (*n* = 129) and a 10.8% in participants with at least one known risk factor for TB exposure (*n* = 185).

The QFT-CLIA platform has strong advantages compared to QFT-ELISA. The automatized system is a random access platform with a turnaround time of less than 1 hour that allows the processing of a large volume of samples, limiting variability, ensuring reproducibility, and reducing laboratory work (8–10). QFT-LF, on the other hand, had even more potential accessibility advantages, although it is no longer marketed, and in our findings, the overall agreement with QFT-ELISA was lower (76.4%) than previously reported (94.9%–98.8%) (11, 12, 14, 15).

As a strength of this study, samples were processed fresh and in parallel, and blind to the outcome, as opposed to other studies where sample selection was based on known previous QFT-ELISA (4, 9, 10) or QFT-CLIA results (6) (positive, negative, or indeterminate). Additionally, the samples of this study belonged to a prospective cohort of TB household contacts (mostly all BCG vaccinated), a population target for the IGRA tests, including individuals from countries with low and high TB incidence. As a study limitation, we used only two different lots for QFT-ELISA testing introducing less variability in the assay, compared to QFT-CLIA which was performed using a total of six different lots over time.

In conclusion, our results contribute to the evaluation of the different QFT platforms commercially available and highlight that these may not be directly interchangeable as their concordance is low for borderline positive results, with an overall 16.6% higher positivity rate for QFT-CLIA among TB contacts. Whether this higher positivity rate corresponds to true infections or false positive results due to the observed inflated IFN-γ values by the QFT-CLIA platform, requires an independent diagnostic test validation study.

## MATERIALS AND METHODS

### Patient selection

Participants belonged to a prospective cohort study of household contacts (≥6 years old) of patients with active pulmonary TB disease from three Health Services areas of the Metropolitan Region in Santiago, Chile (NCT04938596). Blood sampling for IGRA testing was done at enrollment and repeated at a 12-week follow-up if IGRA results were negative at baseline testing (based on QFT-ELISA result). Samples selected for the QFT-ELISA vs QFT-CLIA comparative analysis belonged to consecutive participants from this cohort, recruited between May 2023 and November 2023, while samples selected for the QFT-ELISA vs QFT-LF analysis corresponded to stored frozen samples from consecutive participants of the cohort recruited between July 2022 and February 2023.

### Interferon-γ measurement

Blood samples were directly collected in the QuantiFERON-TB Gold Plus Blood Collection Tubes and transported at room temperature within less than 16 hours for processing to the Laboratory of Microbiology from the Red de Salud UC—CHRISTUS, Santiago, Chile, complying with the sample stability instructions. All blood samples were incubated for 16–20 hours at 37°C and then centrifuged.

For QFT-ELISA vs QFT-CLIA comparison, after centrifugation, the supernatants from the QFT tubes were stored at 4°C for 4 days on average (range: 0–17 days). Before processing, samples were allowed to reach room temperature, then re-centrifuged and processed manually according to the manufacturer’s instructions. Both assays were performed on the same day, simultaneously. For a subset of these samples, remainings of the supernatants from the QFT tubes were stored at −80°C to perform an additional stability analysis of the IFN-γ levels after a 3-month freezing period. After a mean time of 87 days (range: 70–119 days) frozen samples were thawed at room temperature for at least 60 minutes for equilibration, and then re-analyzed by both QFT-ELISA and QFT-CLIA platforms as previously described.

The QFT-ELISA vs QFT-LF comparison was done with frozen supernatants that had been stored at −80°C in QFT tubes. Supernatants were thawed after a mean time of 90 days (range: 3–175 days) at room temperature for at least 60 minutes; then re-centrifuged and processed as usual.

For QFT-ELISA IFN-γ concentration, sample measurements were determined with the calibration curve performed in each assay; for QFT-CLIA, they were automatically determined by the LIAISON automated system, and results interpretation for all assays was performed according to the manufacturer’s instructions. To simplify, for QFT-ELISA and QFT-CLIA, TB1 minus Nil, and TB2 minus Nil will be referred to as TB1 and TB2, respectively. The borderline range was defined as values of IFN-γ between 0.35 and 0.99 IU/mL (20). For QFT-LF, the results are provided as negative or positive, and quantitatively as TTR in seconds.

Additionally, the human IFN-γ standard included in the QFT-ELISA kit was reconstituted and a 7-point standard curve was constructed. Serial dilutions (4, 2, 1, 0.5, 0.25, 0.125, and 0 IU/mL) were made with distilled water and simultaneously analyzed with both QFT-ELISA and QFT-CLIA platforms. The two assays were performed in triplicate twice.

### Statistical analysis

Cohen’s kappa (κ) (21) and Spearman’s (r_S_) correlation coefficient were calculated. A segmented regression model was built for IFN-γ levels obtained from the TB1 and TB2 tubes with the QFT-ELISA and QFT-CLIA platforms, while a polynomial regression was constructed for the IFN-γ levels from the QFT-ELISA TB2 tube and the time to result for QFT-LF. Comparisons of IFN-γ levels obtained with QFT-ELISA vs QFT-CLIA were done with the Wilcoxon paired test and a Bland-Altman plot. For quantitative and correlation analysis only, samples with IFN-γ results >10 IU/mL were excluded, given that they were not further quantified. For all analyses, two-sided *P*-values < 0.05 were considered statistically significant. All analyses were performed using R (version 4.3.1) with the RStudio interface (version 1.3.1073) (22, 23).

## SUPPLEMENTAL MATERIAL

Fig. S1 spectrum.00469-24-s0001.tiff Comparison of IFN-γ levels obtained with simultaneous QFT-ELISA and QFT-CLIA in fresh samples (only QFT-CLIA-negative results included).

Fig. S2 spectrum.00469-24-s0002.tiff IFN-γ differences between QFT-CLIA and QFT-ELISA measurements for all samples.

Fig. S3 spectrum.00469-24-s0003.tiff Comparison of IFN-γ levels obtained with QFT-ELISA and QFT-CLIA in fresh vs frozen samples (only QFT-CLIA-positive results included).

ASM does not own the copyrights to Supplemental Material that may be linked to, or accessed through, an article. The authors have granted ASM a non-exclusive, world-wide license to publish the Supplemental Material files. Please contact the corresponding author directly for reuse.
