# Supplementary figures and images for: Evaluation of concordance of new QuantiFERON-TB Gold Plus platforms for Mycobacterium tuberculosis infection diagnosis in a prospective cohort of household contacts
Source: Microbiol Spectr. 2024 Jul 8;12(8):e00469-24. doi: 10.1128/spectrum.00469-24 (PMC11302262; doi:10.1128/spectrum.00469-24)

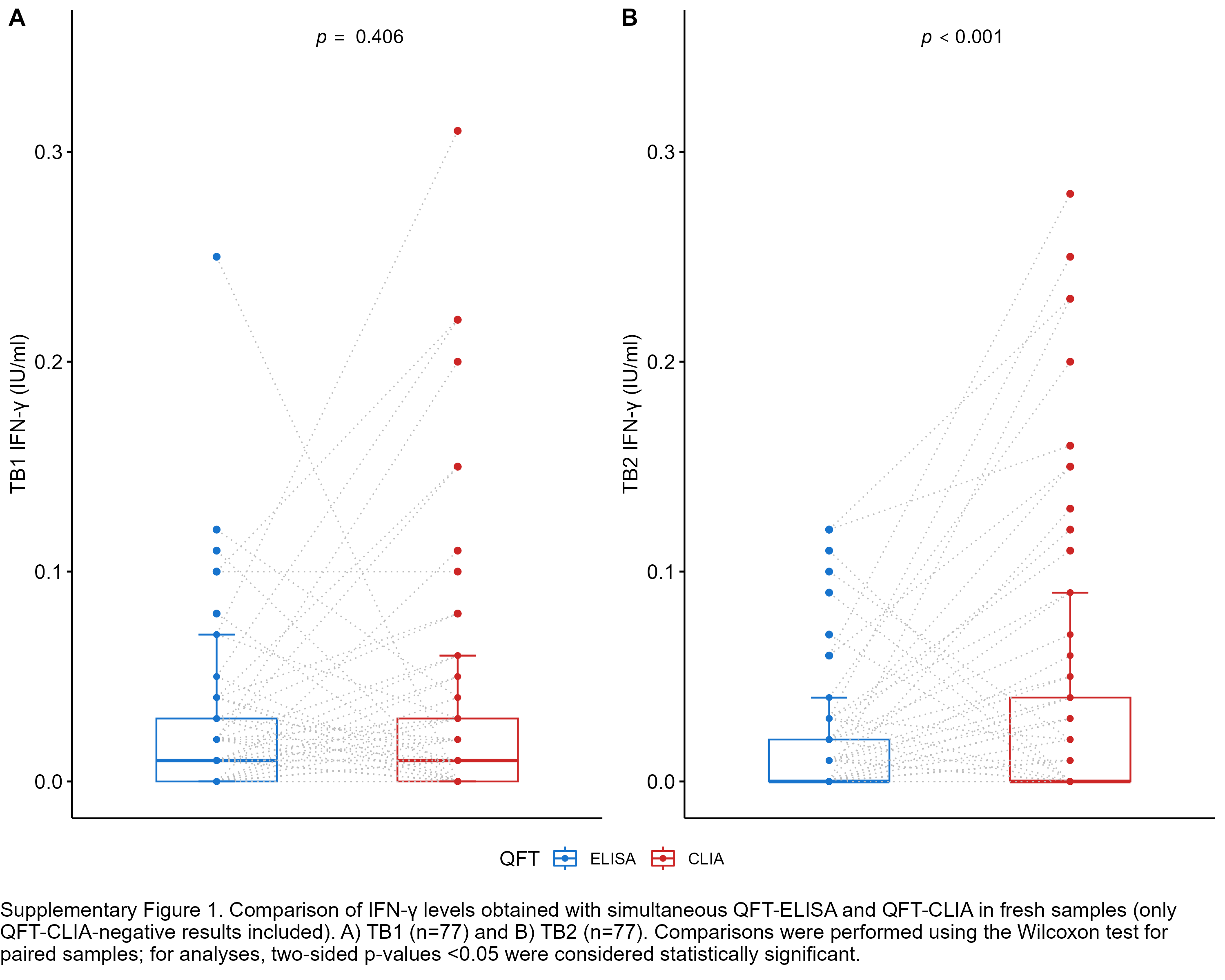

Supplement: Fig. S1 — Comparison of IFN-γ levels obtained with simultaneous QFT-ELISA and QFT-CLIA in fresh samples (only QFT-CLIA-negative results included). [file spectrum.00469-24-s0001.tiff]

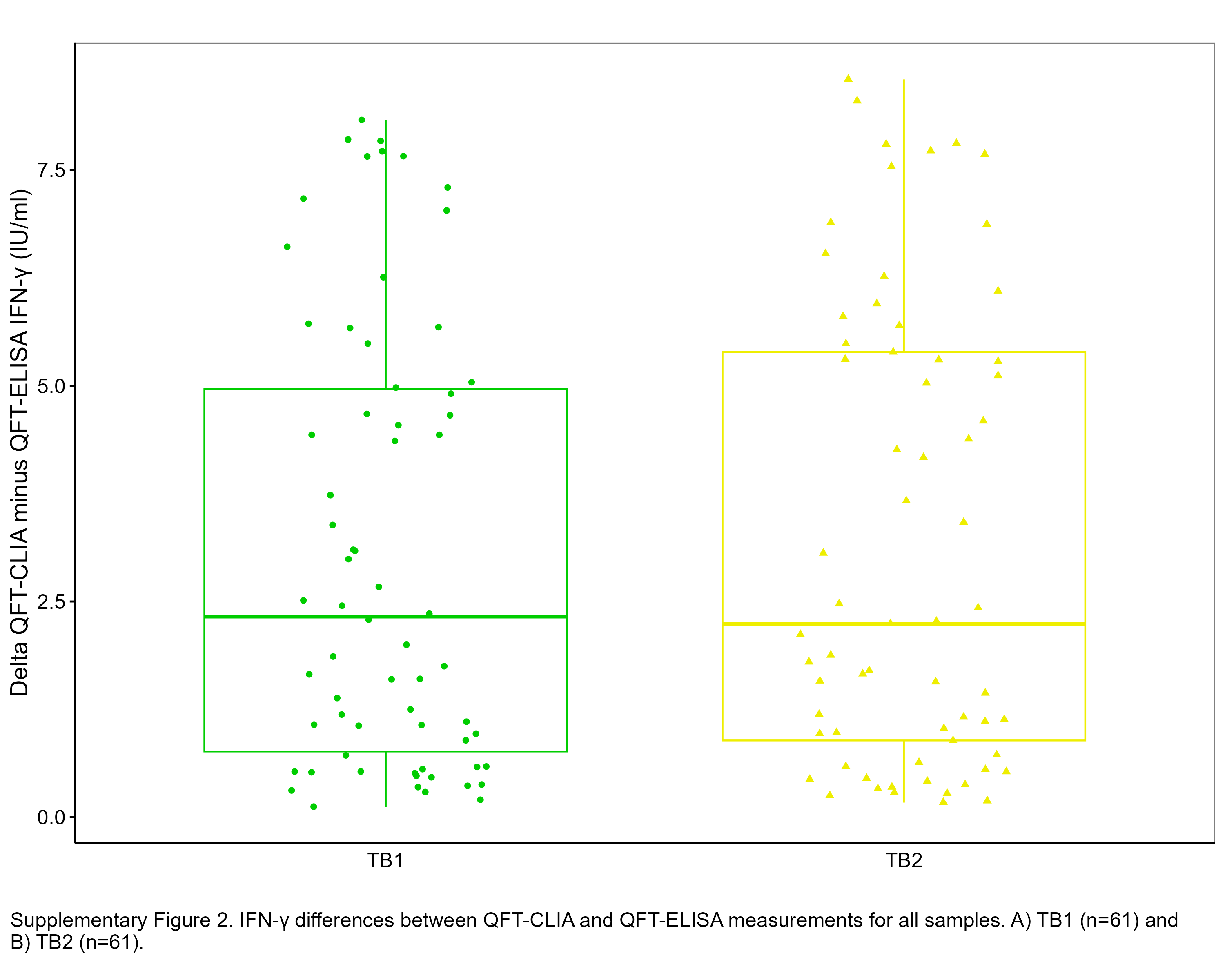

Supplement: Fig. S2 — IFN-γ differences between QFT-CLIA and QFT-ELISA measurements for all samples. [file spectrum.00469-24-s0002.tiff]

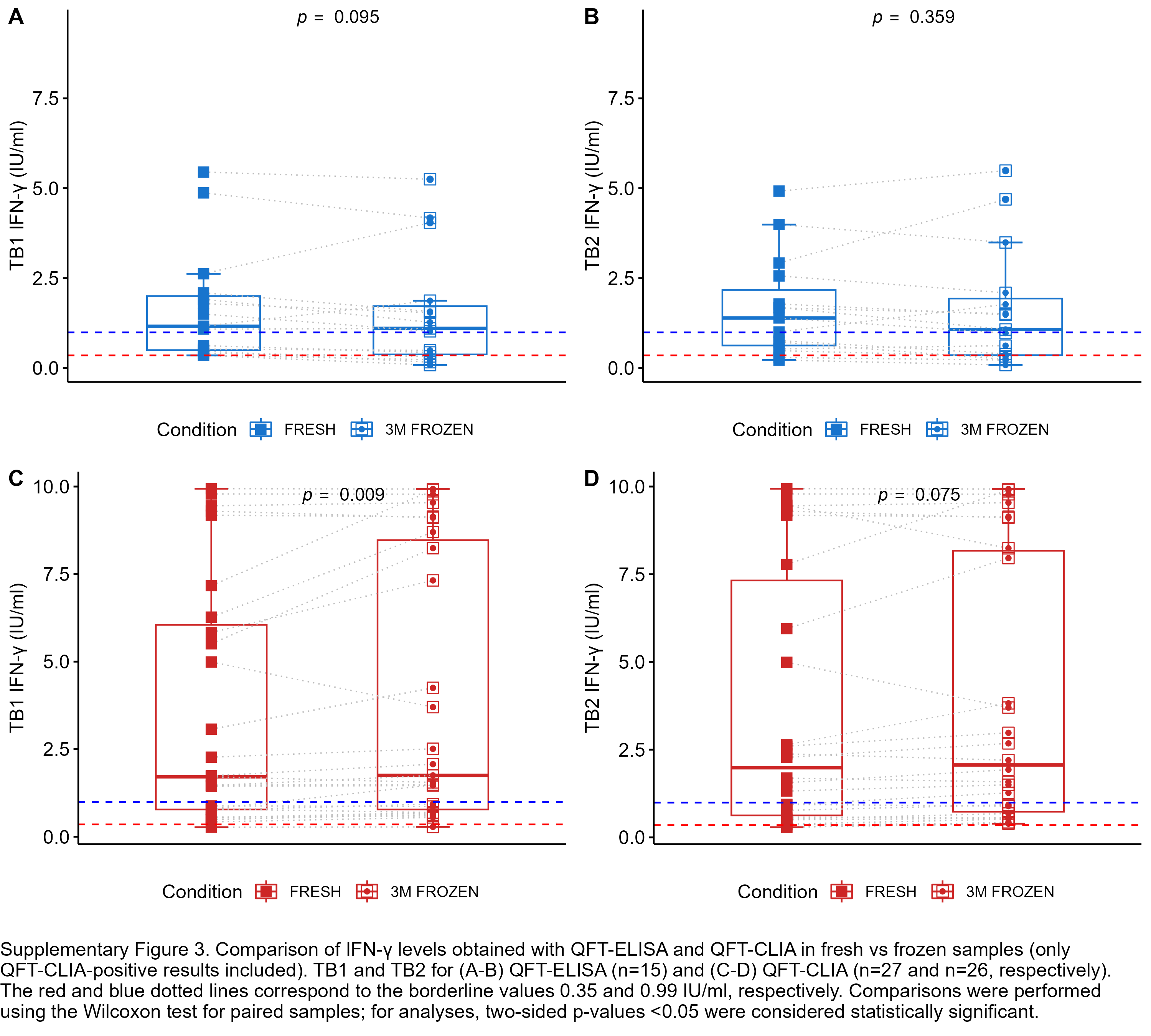

Supplement: Fig. S3 — Comparison of IFN-γ levels obtained with QFT-ELISA and QFT-CLIA in fresh vs frozen samples (only QFT-CLIA-positive results included). [file spectrum.00469-24-s0003.tiff]
